# Complete Genome Sequence of *Listeria monocytogenes* Lm60, a Strain with an Enhanced Cold Adaptation Capacity

**DOI:** 10.1128/genomeA.01248-14

**Published:** 2014-12-04

**Authors:** Taurai Tasara, Thomas Weinmaier, Jochen Klumpp, Thomas Rattei, Roger Stephan

**Affiliations:** a Institute for Food Safety and Hygiene, Vetsuisse Faculty, University of Zurich, Zurich, Switzerland; b Division of Computational Systems Biology, Department of Microbiology and Ecosystem Science, Faculty of Life Sciences, University of Vienna, Vienna, Austria; c Institute of Food, Nutrition and Health, ETH Zurich, Zürich, Switzerland

## Abstract

The complete genome sequence of *Listeria monocytogenes* Lm60, a fast cold-adapting serotype 1/2a human isolate, is presented.

## GENOME ANNOUNCEMENT

*Listeria monocytogenes* is an important food-borne pathogen associated with serious illness and high mortality in those with weakened immunity (1). In particular the capacity of this bacterium to grow on cold preserved foods is a significant public health and food safety concern (2, 3). Strains with an enhanced ability to adapt and grow at refrigeration temperatures pose heightened risks, since they might multiply more efficiently reaching levels required for human infection within the shelf life of some cold preserved foods (4). At present there is little known about the molecular and genetic mechanisms responsible for the enhanced cold adaptability phenotypes displayed by some *L. monocytogenes* strains compared to others (3, 5–9). Lm60 is a fast cold-adapting strain that was isolated from a sporadic human listeriosis case in Switzerland. This strain, when transferred from 37°C to 4°C, requires a significantly shorter lag time for cold adaptation resuming growth after about 9 h compared to between 70 and 200 h required by the majority of other *L. monocytogenes* strains (9).

We present here the complete genome sequence of *L. monocytogenes* Lm60. Genomic DNA was isolated from Lm60 and subjected to single-molecule real-time sequencing on a Pacific Biosciences RS2 device (10-kb insert library, P4/C2 chemistry) at the Functional Genomics Centre at the University of Zurich. Sequencing resulted in 27, 714 sequence reads (48-fold genome coverage) with an average length of 5,854 kb. The Lm60 genome was assembled *de novo* using the SMRT Analysis 2.1.1 software and HGAP3 algorithm to a single chromosome of 2,989,591 bp in size with a G+C content of 38%. Gene prediction and annotation was carried out using the NCBI Prokaryotic Genomes Automatic Annotation Pipeline.

The Lm60 genome contains a total of 3,002 genes including 2,904 coding sequences (CDS), 12 pseudogenes, 67 tRNA genes, and 6 16S-5S-23S operons. Using the Phage search tool (PHAST) (10), the Lm60 genome was predicted to harbor two incomplete prophages located at positions 1,913,313 to 1,936,288 (22,976 bp) and 2,815,070 to 2,863,134 (48,065 bp), as well as two-phage-like regions at positions 771,018 to 824,162 (53,145 bp) and 2,591,434 to 2,643,286 (51,853 bp). An *in silico* multilocus sequence type (MLST) analysis was performed using the MLST targets (*abcZ*, *cat*, *dat*, *lhkA*, *bglA*, *dapE*, and *ldh*) and Lm60 was assigned to sequence type (ST) 551 (11) (http://www.pasteur.fr/recherche/genopole/PF8/mlst/index.html).

The availability of the genome sequence from this strain will provide insight into possible genetic mechanisms associated with enhanced cold stress resistance among fast cold-adapting *L. monocytogenes* strains.

### Nucleotide sequence accession number.

The complete Lm60 genome has been deposited in GenBank under the accession no. CP009258.

## References

[1] Allerberger F Wagner M 2010 Listeriosis: a resurgent foodborne infection. Clin. Microbiol. Infect. 16:16–23. 10.1111/j.1469-0691.2009.03109.x. 20002687

[2] Walker SJ Archer P Banks JG 1990 Growth of *Listeria monocytogenes* at refrigeration temperatures. J. Appl. Bacteriol. 68:157–162. 10.1111/j.1365-2672.1990.tb02561.x. 2108109

[3] Junttila JR Niemelä SI Hirn J 1988 Minimum growth temperatures of *Listeria monocytogenes* and non-haemolytic listeria. J. Appl. Bacteriol. 65:321–327. 10.1111/j.1365-2672.1988.tb01898.x. 3146567

[4] Nufer U Stephan R Tasara T 2007 Growth characteristics of *Listeria monocytogenes*, *Listeria welshimeri* and *Listeria innocua* strains in broth cultures and a sliced Bologna-type product at 4 and 7 degrees C. Food Microbiol. 24:444–451. 10.1016/j.fm.2006.10.004. 17367677

[5] Lianou A Stopforth JD Yoon Y Wiedmann M Sofos JN 2006 Growth and stress resistance variation in culture broth among *Listeria monocytogenes* strains of various serotypes and origins. J. Food Protect. 69:2640–2647. 10.4315/0362-028x-69.11.2640 17133807

[6] Barbosa W Cabedo B Wederquist H Sofos JN 1994 Growth variation among species and strains of Listeria in culture broth. J. Food Protect. 57:765–769. 10.4315/0362-028X-57.9.765 31121799

[7] Begot C Lebert I Lebert A 1997 Variability of the response of 66 *Listeria monocytogenes* and *Listeria innocua* strains to different growth conditions. Int. J. Food Microbiol. 14:403–412. 10.1006/fmic.1997.0097.

[8] Arguedas-Villa C Kovacevic J Allen KJ Stephan R Tasara T 2014 Cold growth behaviour and genetic comparison of Canadian and Swiss *Listeria monocytogenes* strains associated with the food supply chain and human listeriosis cases. Food Microbiol. 40:81–87. 10.1016/j.fm.2014.01.001. 24549201

[9] Arguedas-Villa C Stephan R Tasara T 2010 Evaluation of cold growth and related gene transcription responses associated with *Listeria monocytogenes* strains of different origins. Food Microbiol. 27:653–660. 10.1016/j.fm.2010.02.009. 20510784

[10] Zhou Y Liang Y Lynch KH Dennis JJ Wishart DS 2011 PHAST: a fast phage search tool. Nucleic Acids Res. 39:W347–W352. 10.1093/nar/gkr485. 21672955 PMC3125810

[11] Ragon M Wirth T Hollandt F Lavenir R Lecuit M Le Monnier A Brisse S 2008 A new perspective on *Listeria monocytogenes* evolution. PLoS Pathog. 4:e1000146. 10.1371/journal.ppat.1000146. 18773117 PMC2518857

